# Prediction Models for Early Identification of Overweight and Obese Children—A National Study

**DOI:** 10.3390/nu18030441

**Published:** 2026-01-29

**Authors:** Irit Lior Sadaka, Itamar Grotto, Yair Sadaka, Roni Eilenberg, Assaf Peleg, Dan Greenberg

**Affiliations:** 1 Department of Health Policy and Management, School of Public Health, Faculty of Health Sciences, Ben-Gurion University of the Negev, Be’er-Sheva 84105, Israel; 2 Department of Epidemiology, Biostatistics, and Community Health Sciences, Faculty of Health Sciences, Ben-Gurion University of the Negev, Be’er-Sheva 84105, Israel; grotto@bgu.ac.il; 3 INSPIRE Institute, Faculty of Health Sciences, Ben-Gurion University of the Negev, Be’er-Sheva 84105, Israel; 4 Timna Initiative, Ministry of Health, Jerusalem 9446724, Israel

**Keywords:** infant growth, childhood obesity, early risk prediction, growth monitoring, anthropometric data, WHO growth standards, obesity prevention

## Abstract

Background: Given the importance of early-life intervention in reducing future obesity, screening models that more accurately identify infants at risk are crucial. The aim of this study was to develop models based solely on growth parameters for better predicting childhood overweight than the current WHO growth chart risk prediction. Methods: A retrospective national cohort study was conducted among children born in Israel between August 2014 and June 2016, followed for at least 18 months. Machine learning models were generated to predict childhood overweight. Three models for 0–3, 3–6, and 6–12 months were generated. These models were compared with the current WHO growth chart predictions. The outcome was defined as overweight in early childhood, based on weight-for-length (or weight-for-height) ≥97th percentile, according to WHO standards, measured at 18–36 months of age. Results: Overall, 198,503 children were included, and 150,572, 146,584, and 149,628 infants were included in Models 1, 2, and 3, respectively, with an average target age of two years. The models demonstrated high predictive performance (AUC) for the 0–3 months’ model (0.76 [95% CI: 75 to 76.9%]), for the 3–6 months’ model (0.822 [95% CI: 81.3 to 83.0%]) and for 6–12-month-old infants (0.872 [95% CI: 86.6 to 87.8%]). The first two models better predict the risk of early childhood overweight than the current WHO growth chart prediction. Conclusions: These models are unique in that they are based on growth parameters, usually screened at early childhood worldwide, and can be implemented in any system collecting growth measurements of infants, providing better risk prediction than the current WHO growth charts. A web calculator is provided.

## 1. Introduction

The worldwide prevalence of adult obesity has nearly tripled over the last four decades [1], and the prevalence of obesity in infants, children, and adolescents is rapidly growing [2]. Childhood and adolescent obesity are associated with several co-morbidities, including abnormalities in the endocrine, cardiovascular, gastrointestinal, pulmonary, orthopedic, neurologic, dermatologic, and psychosocial systems [3,4]. Overweight in infancy is an independent risk factor for obesity in childhood, adolescence, and adulthood [5]. There is strong evidence to support the beneficial effects of child obesity prevention and intervention programs on later obesity, and the earlier the intervention is performed, the greater the effect measured [6]. Prominent health organizations such as the World Health Organization (WHO), the Institute of Medicine (IOM) in the United States, and the American Academy of Pediatrics (AAP) call for early intervention to reduce childhood obesity [7,8].

One of the key elements of child preventive medicine programs worldwide is the use of the WHO Child Growth Standards [9] in every well-child visit. This instrument is used for early growth screening and is the main suggested screening tool for overweight and obesity. According to the WHO, the definition of overweight for children under 5 years is a weight-for-length (or weight-for-height) greater than 2 standard deviations (97th percentile) above the WHO Child Growth Standards median [1]. Children between the 85th and 97th percentile lines on the growth chart are defined as at risk for overweight [10]. As part of efforts to prevent childhood obesity, the IOM recommends that healthcare professionals consider children’s attained weight-for-length at or above the 85th percentile as a risk factor in assessing which young children are at the highest risk for later obesity [10]. Given the importance of early intervention in preventing future overweight and obesity, it is crucial to use the best screening models to identify infants at risk. The main objective of this study was to develop prediction models for early identification of overweight and obese children in a large national cohort of infants in Israel. We then compared these models to the current suggested WHO worldwide screening paradigm.

## 2. Methods

This retrospective cohort study included all children born in Israel between August 2014 and June 2016 who were followed for at least 18 months and had available records in our database.

### 2.1. The Database

Preventive care and health promotion services for pregnant women and early childhood are provided in Israel through the healthcare mother and child care centers (“Tipat Halav”). They provide free health and medical screening for pregnant women and for child growth and development for all Israeli residents [11]. The objectives of the services provided are the prevention of infectious diseases through immunization, the early detection of health problems through routine growth and development examinations, and health education. Recommended visits are at post-birth and at 1, 2, 4, 6, 9, 12, 18, 24, 36, 48, and 60 months afterward. The healthcare staff includes nurses and physicians who have been specially trained in children’s public health [12].

There are about 1000 Tipat Halav clinics across Israel, operated by various parties. The Ministry of Health (MOH) and some local municipalities cover and operate approximately 80% of the clinics and share a single, comprehensive, computerized database used in this study. Other children are also served free of charge by additional clinics, mainly operated by the four Health Maintenance Organizations (HMOs) operating in Israel, based on convenience. All the data are entered into a national electronic health record specifically designed for monitoring child growth and development. This database comprises the national database of the Tipat Halav Israeli Screening (THIS) program database. To the best of our knowledge, this is a unique, continuously updated database that contains elaborated data, including family demographics, children’s growth measures, developmental milestones, vaccinations, and age-related health measures and assessments.

### 2.2. Inclusion and Exclusion Criteria

Three models were developed for the following age groups: 0–3, 3–6, and 6–12 months. Separate models were built since more data were available from later visits, and later measurements may be better predictors for childhood overweight and obesity. In all models, we included children with available records in the MOH database who were born between August 2014 and June 2016 and had at least 18 months of follow-up. Included children were only those with at least one weight-for-height measurement at 18–36 months. Also, each model included children with at least one weight-for-height measurement record for the specific time period (0–3, 3–6, and 6–12 months for the first, second, and third models, respectively). We excluded preterm infants because we believe this population warrants separate, careful analysis.

### 2.3. Model Variables

For each of the three models, the *dependent variable* (target variable) was defined as an indicator variable if the last visit measurement of the weight-for-height (or weight-for-length) percentile at 18–36 months was equal to or greater than the 97th percentile.

Various *independent variables* (predictors) were extracted from the THIS database, including demographics (age, gender, religion), birth variables (birth weight, birth type, gestational week, APGAR score), mother’s characteristics (mother’s age, education, depression status based on the Edinburgh Postnatal Depression Scale (EPDS) [13], family status, immigration status), child growth measurements (weight, length or height, weight-for-length, and head circumference percentiles), nutrition (nursing period, vitamin-D supplementation, iron supplementation, age at starting formula, formula type, age at starting water).

Because trends in growth measurements may be crucial for predicting the risk of overweight, we created new variables based on children’s growth measurements. These variables included the linear slope of the last weight-for-length percentile measurements, linear slope of the last weight percentile measurements, difference between the last and first weight-for-length percentiles divided by the age difference, ratio between the head circumference percentile and the weight-for-length percentile, ratio between the last two weight-for-length percentiles, maximum growth measurement percentile (weight-for-length and weight percentiles) throughout the relevant model period (0–3, 3–6, 6–12 months), and the higher measurement of the last two or more weight-for-length measurements throughout the relevant model period (0–3, 3–6, 6–12 months).

Since all variables included in the model are routinely recorded in the THIS program database regardless of the child’s weight, the outcome and its predictors are assessed blindly in the current study.

### 2.4. Machine Learning Algorithms

Machine learning is considered a method that can augment the diagnosis of chronic diseases. The key distinction between traditional approaches and machine learning is that in machine learning, a model learns from examples rather than being programmed with rules. Using algorithms for learning from observations, computers then determine how to perform the mapping from features to labels to create a model that will generalize the information, enabling a task to be performed correctly with new, never-before-seen inputs [14]. Feature selection was performed using a Random Forest machine learning algorithm. Random Forest algorithms determine the variables’ importance by calculating the relative influence of each variable: this determination is based on whether that variable was selected to split the tree further during the tree building process, and on how much the squared error at the split nodes was reduced, aggregated over all trees [15,16].

Variables with high relative importance were included in the XGBoost algorithm. XGBoost is a supervised learning algorithm that uses a boosting technique to produce accurate models. Boosting is an ensemble learning technique that builds many models sequentially, with each new model attempting to correct for the deficiencies of the previous model. In tree boosting, each new model that is added to the ensemble is a decision tree [17]. Feature importance derived from the Random Forest model guided the selection of predictors included in the XGBoost models. The final set of variables was determined based on model performance stability and overfitting avoidance, rather than a predefined importance threshold. Hyperparameter tuning for the XGBoost models was performed using a random search with 5-fold cross-validation. The final model configuration included a tree depth of three and 200 trees, as these settings provided stable predictive performance without excessive model complexity.

Missing values were not imputed during model training; the machine-learning algorithms handled them natively. During tree building, split decisions for each node were optimized by minimizing the loss function, while allowing missing values to be treated as a separate category that could be assigned to either branch. This approach does not assume a specific clinical or socioeconomic reason for missingness, but rather allows the algorithm to determine whether patterns of missing data contribute to prediction performance, without implying causality or introducing predefined bias.

The model population was randomly divided into a training set (70%) and a test set (30%). Model construction was performed using the training set, and the test set was used for model evaluation. This approach was chosen to reflect real-world implementation scenarios. Future studies may explore additional internal validation strategies to further assess robustness.

### 2.5. Model Evaluation

Model evaluation was performed on the test set. The model risk scores were calculated for each individual in the test set. Then each individual was ranked by its risk score into percentiles (100 = high risk, 1 = low risk). We measured specificity, sensitivity, positive predictive value (PPV), and negative predictive value (NPV) to assess the model’s performance. Lift and AUC were also calculated. The performance of each of the three models was compared to that currently used by WHO for weight-for-length prediction. For comparison, WHO-based screening was defined using weight-for-length percentiles measured during the corresponding infant age windows, with overweight defined as ≥97th percentile according to WHO standards. This approach reflects current screening practice rather than a predictive model and was used as a benchmark for evaluating model performance.

### 2.6. Statistical Analyses

Statistical analyses were performed using Python version 3.6.7. Random Forest and XGBoost models were constructed using the H_2_O package version 3.22 [18].

The study follows transparent reporting of a multivariable prediction model for individual prognosis or diagnosis, the TRIPOD statement.

### 2.7. Ethics Declarations

All analyses were carried out in accordance with relevant guidelines and regulations. The study protocol was approved by the Soroka University Medical Center Institutional Ethics Committee (MHC-0014-19; 5 July 2018). Informed consent was waived owing to the use of de-identified data.

## 3. Results

**Figure 1:**
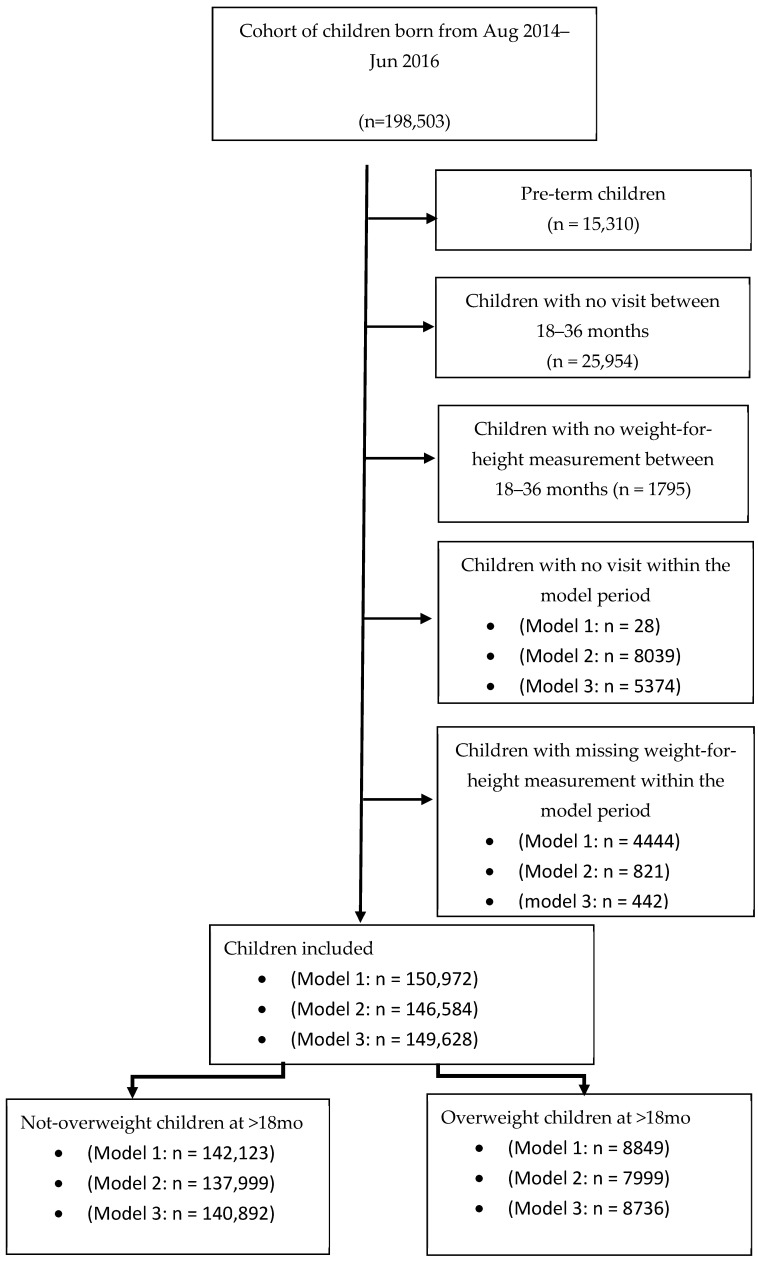
The models’ cohorts: Sample sizes differ across models due to age-specific inclusion criteria and the availability of measurements within each age window.

A total of 198,503 children born between August 2014 and June 2016 who had relevant records in the THIS national program database were screened (approximately 60% of the children born in Israel during this period). Altogether, 15,310 pre-term children (gestational age < 37 weeks), children lacking at least one visit (*N* = 25,954), or with a missing value of the weight-for-height measurement between ages 18 and 36 months (*N* = 1795) were excluded from the analysis. Children lacking at least one visit during the specific model period (0–3 months: *N* = 28, 3–6 months: *N* = 8039, and 6–12 months: *N* = 5374) and children with missing weight-for- length (or weight-for-height) measurements were also excluded (0–3 months: *N* = 4444, 3–6 months: *N* = 821, and 6–12 months: *N* = 442). Overall, 150,572, 146,584, and 149,628 children were included in Models 1, 2, and 3, respectively (Figure 1). Main cohort characteristics are summarized in Table 1.

### 3.1. Prediction Equations

#### 3.1.1. Model for 0–3 Months

**Figure 2:**
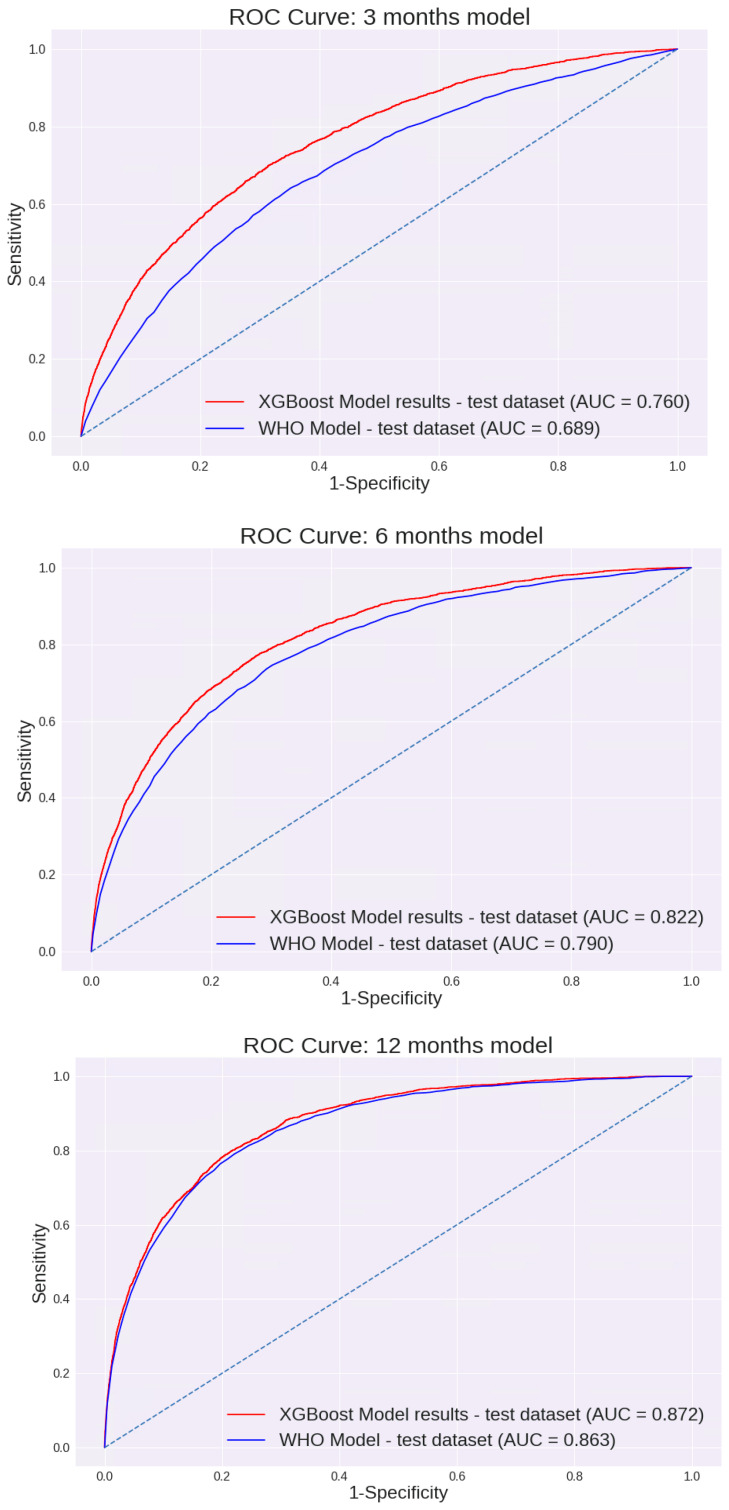
AUC ROC Curves of the models: ROC curves compare model-based prediction to WHO weight-for-length percentile–based screening (≥97th percentile) for each age window.

Feature selection was performed using a Random Forest machine learning algorithm. Of all variables entered into the model, 15 were selected for further analysis using an XGBoost model (Table 2). The performance of the 0–3-month model for different cut points is presented in Table 3. This model prediction was compared to the prediction made using the WHO weight-for-length percentile. As demonstrated in Figure 2, the AUC of this model was 0.760 [95% CI: 75.4 to 77.3%]. These values were substantially higher than the AUC calculated using the current WHO model based on the weight-for-length percentile only: 0.689 [95% CI: 67.9 to 69.9%].

#### 3.1.2. Model for 3–6 Months

Of the variables described, 14 were selected for further analysis using the XGBoost model (Table 2). The performance of the 3–6-month model across different cut points is shown in Table 3. The AUC of this model was 0.822 [95% CI: 81.3 to 83.0%]. Again, these values were significantly higher than the AUC calculated using the current WHO model based on the weight-for-length percentile: 0.79 [95% CI: 78.0 to 79.9%]. (*p* < 0.05). The AUC curves are presented in Figure 2.

#### 3.1.3. Model for 6–12 Months

Of all variables entered into the model, 9 were selected for further analysis using the XGBoost model (Table 2). Unlike previous models, the difference between the AUC values of the two models was not significantly different; the AUC of our model was 0.872 [95% CI: 86.6 to 87.8%], and the AUC using the current WHO model was 0.863 [95% CI: 85.6 to 87%] (Figure 2). The sensitivity, specificity, PPV, and NPV of each model are shown in Table 3. Interestingly, although many different variables were entered in each of the models, including demographic variables, birth variables, mothers’ characteristics, nutrition, and child growth measurements, all models were eventually based solely on growth measurement parameters. The only exception was the age when formula was added to or replaced breast milk (“age at starting formula”) for the first 0–3 months’ model, which made a minimal contribution. The performance of the first model, based solely on growth parameters excluding this variable, was examined. The AUC was 0.76 [95% CI: 75 to 76.9%], a reduction of less than 0.5% compared with the model that included this variable. In all other models, growth measurements were the only final variables included.

#### 3.1.4. Validity of the Models for Different Target Ages

Further analyses were conducted for each model when the target weight-for-height percentile was measured at age 18–24 months or 24–36 months. According to the analysis, the three models show similar trends for both earlier and later target ages (Table 1, Appendix A).

#### 3.1.5. Validity of the Models in Different Populations

To examine whether this model’s performance was valid across populations of different ethnic backgrounds, performance was evaluated separately for Muslim (27% of the cohort) and Jewish (66% of the cohort) infants. No differences were found between these populations. For example, using the 3–6-month model, the AUC of Muslim infants was 0.817 [95% CI: 80.1 to 83.5%], and for Jewish infants, 0.826 [95% CI: 81.6 to 83.7%], with no significant difference between these two subpopulations.

## 4. Discussion

Three different models were built for 0–3-, 3–6-, and 6–12-month-old infants. Our models demonstrate higher AUC performance than the WHO Child Growth Standards for the 0–3- (0.760 vs. 0.689; *p* < 0.05) and 3–6-month (0.822 vs. 0.79; *p* < 0.05) models. However, for 6–12 months, we could not establish a model with significantly better performance than the WHO model (0.872 vs. 0.863). The model’s AUC was higher for older infants (6–12 months > 3–6 months > 0–3 months). This might, in part, be due to additional measurements being available for the older infants. It is also possible that the higher performance observed in older infants reflects the shorter interval between the age at prediction and the age at which overweight was assessed. To address this, we evaluated model performance separately for target measurements at 18–24 months and 24–36 months. Model performance remained stable across these age ranges, with no significant differences in AUC, suggesting robustness across the toddler age window.

In this study, we assessed the contribution of multiple variables, including demographics, pregnancy and birth variables, mothers’ characteristics, and nutrition. However, the most significant predictors were based on growth measurements commonly conducted during infant surveillance visits. We assume that growth measurements at birth and throughout development most accurately reflect the final effects of all relevant factors that contribute to a child’s risk of overweight and obesity. Although there are many genetic, pregnancy, birth-related, or later early-years’ risk factors for overweight and obesity, they will have a major effect on the child’s growth measurements used for predicting overweight. Since growth measurements are recommended to be routinely measured at well-baby or vaccination visits, these data should be available for parents and healthcare providers [19,20].

This study builds a machine learning clinical prediction model for early childhood overweight using a comprehensive national cohort, including a multivariable database of healthy children. This model can be easily implemented in systems that collect growth measurements, providing better prediction of the risk of early childhood overweight than the current WHO prediction, using the same commonly collected measurements at no additional effort. Link for THIS early childhood overweight risk calculator is provided here (https://www.yeled-dev.org/risk-of-overweight-calculator/ (accessed on 8 January 2026)).

Although clinical prediction models are increasingly prevalent [21,22], few studies have focused on the prediction of overweight or obesity in early childhood [23,24,25,26,27,28,29,30]. Most models are based on small to medium samples rather than a national cohort, and none provide easily implemented tools based solely on growth measurements.

Using these suggested models may improve infant screening for overweight. According to the WHO assessments, if current trends continue, the number of overweight or obese infants and young children under five years of age globally will increase to 70 million by 2025 [1]. Using our 0–3-month model (threshold selected—5% with the highest score), out of 1,000,000 newborns per year, it will be possible to detect 4500–5000 more overweight or obese children than using WHO prediction (over 97th percentile) (based on the sensitivity difference), while 4500–5000 more children will be accurately classified as being at no risk for overweight (based on the specificity difference). Thus, this model is important when targeted interventions are considered for infants at risk for overweight and obesity, given the need for directing resources to the actual populations in need. Different thresholds are provided to ensure an appropriate match between the intervention’s resources and costs and the relevant target population. For infants aged 6–12 months, the predictive performance of the proposed model was comparable to that of the WHO growth chart–based approach. While this limits the incremental statistical advantage of the model in this age group, its potential value lies in other aspects, including automation, consistency across age-specific models, and ease of integration into electronic medical record–based surveillance systems. Importantly, the main clinical added value of the proposed approach is observed during the first six months of life, when the models demonstrate clear superiority over the WHO growth charts and when early preventive interventions may have the greatest impact.

As with any screening tool, false-positive and false-negative classifications must be considered in a clinical context. In particular, identifying an infant as being at increased risk for overweight at an early age may raise concerns regarding potential parental anxiety. It is important to emphasize that the proposed models are intended for risk stratification rather than diagnosis, and should be used to guide supportive, non-stigmatizing nutritional counseling and follow-up. When applied appropriately, the benefits of early identification and targeted prevention may outweigh the potential psychosocial risks associated with false-positive results.

There are some limitations to our study. The research was conducted only with children who attended Tipat Halav clinics. Children of parents not compliant with follow-ups were excluded. The Israeli immunization coverage in Tipat Halav centers ranges between 94% and 99%, reflecting the high compliance with this service [31].

Also, the study was conducted only with children from Israel. However, the Israeli population is multicultural, and although a wide range of demographic, maternal, pregnancy, and delivery-related variables were initially included in the models, none were retained in the final models. The final prediction models were based exclusively on routinely collected growth parameters, which are universally measured in early childhood surveillance programs worldwide. This suggests that model performance relies primarily on growth trajectories rather than population-specific characteristics, supporting the potential external validity of the proposed models. Nevertheless, external validation in other countries and healthcare systems is required to confirm their generalizability.

Also, there are further variables omitted in this study that might further improve the model’s prediction, such as mother’s Body Mass Index (BMI) prior to pregnancy, maternal smoking status during pregnancy and paternal BMI [30]; however, these variables are not commonly available in any electronic medical records (EMRs), and thus, models with such variables are not feasible for easy implementation within these systems. Finally, although discrimination performance was strong, further evaluation of model calibration and clinical utility metrics will be important for future implementation studies.

Overweight is examined in the current study for children up to three years of age. Although this is known to be a crucial period in determining the chances for obesity as adults [5], it is important to further evaluate the model performance for older children and adults. The current models excluded infants with a gestational age of less than 37 weeks. Further specific models should be developed for this population.

## 5. Conclusions

This study introduces innovative models for predicting early childhood overweight and obesity, leveraging commonly screened growth parameters in early childhood. These models outperform the current WHO growth charts in predicting the risk of overweight in young children. Their universal applicability, owing to the routine collection of growth measurements globally, makes them a significant tool in early obesity intervention. Additionally, the study provides a web calculator, enhancing the models’ practical utility and ease of implementation in diverse healthcare settings. From a practical perspective, these models enable early risk stratification using data that are already available during routine well-child visits. This allows healthcare providers to identify infants at increased risk for overweight at a very early age, supporting targeted nutritional counseling, closer growth monitoring, and early preventive interventions. Such an approach may improve decision-making in both clinical and public health settings by focusing resources on infants most likely to benefit from early intervention.

## Figures and Tables

**Table 1:** Cohort characteristics.

Cohort Characteristics	0–3-Month Model n = 150,572	3–6-Month Model n = 146,584	6–12-Month Model n = 149,628
Gender			
Male (%)	51.2%	51.3%	51.2%
Female (%)	48.8%	48.7%	48.8%
Birth weight in kg (Mean, SD)	3.28 (0.44)	3.28 (0.44)	3.28 (0.44)
Gestational age (weeks)	39.4 (1.19)	39.4 (1.19)	39.4 (1.19)
Immigrants (%)	13.1%	13.1%	13.1%
Religion (%)			
Jewish	65.5%	65.2%	65.3%
Muslim	25.9%	26.2%	26.1%
Druse	2.5%	2.5%	2.5%
Cristian Arab	1.8%	1.8%	1.8%
Other	2.5%	2.5%	2.5%
Missing	1.8%	1.8%	1.8%
Mother’s age (Mean, SD)	29.7 (5.6)	29.7 (5.6)	29.7 (5.6)
Type of delivery (%)			
Spontaneous	76.7%	76.6%	76.6%
Assisted	5.5%	5.6%	5.6%
Caesarian	16.6%	16.6%	16.6%
Missing value	1.2%	1.2%	1.2%
Mother’s education (%)			
Elementary schools, middle schools	11.4%	11.1%	11.1%
High school	30.3%	30.3%	30.3%
Academic school	31.4%	31.6%	31.5%
Missing	27%	26.8%	26.9%
Mother’s family status (%)			
Married	92.8%	92.8%	92.8%
Divorced	1.13%	1.13%	1.13%
Widowed	0.05%	0.05%	0.05%
Single	2.92%	2.92%	2.92%
Missing value	3.1%	3.1%	3.1%
Target Child age—months (Mean, SD)	23.8 (4.3)	23.8 (4.3)	23.8 (4.3)
Infant age at each model–months (Mean, SD)	2.16 (0.36)	4.5 (0.53)	8.9 (1.4)
Total number of visits (Mean, SD)	2.3 (0.7)	3.4 (0.73)	5.1 (1.02)

Footnote: Characteristics of children included in the analytic cohort are presented. Sample sizes may vary across age-specific models due to differences in the availability of growth measurements within each time window.

**Table 2:** Final variables selected for each model sorted by relative importance.

Model		Description of Variables Included in the Model
0–3 months	1	Weight-for-height percentile at last measurement
	2	Linear slope of the last three weight percentile measurements
	3	The highest of the last three weight percentile measurements
	4	Weight percentile at the second measurement
	5	Head circumference percentile at last measurement
	6	Leave hospital weight
	7	Weight percentile at last measurement
	8	The highest of the last two weight-for-height percentile measurements
	9	Ratio between birth weight and the pregnancy week at birth
	10	The highest of the last three weight-for-height percentile measurements
	11	Height percentile at last measurement
	12	Birth weight
	13	Head circumference percentile at 2nd measurement
	14	Height percentile at 2nd measurement
	15	Starting formula age (months)
3–6 months	1	Weight percentile at 3rd measurement
	2	Weight percentile at last measurement
	3	Weight-for-height percentile at last measurement
	4	The highest of the last four weight-for-height percentile measurements
	5	The highest of the last four weight percentile measurements
	6	Weight percentile at 2nd measurement
	7	Linear slope of the last four weight percentile measurements
	8	Weight-for-height percentile at 3rd measurement
	9	Ratio between weight-for-height percentile last two measurements
	10	The higher of the last two weight-for-height percentile measurements
	11	Birth Weight
	12	Weight-for-height percentile at 2nd measurement
	13	Head circumference percentile at last measurement
	14	Linear slope of the last four weight-for-height percentile measurements
6–12 months	1	The highest of the last two weight-for-height percentile measurements
	2	The highest of the last six weight-for-height percentile measurements
	3	Weight-for-height percentile at last measurement
	4	Weight percentile at last measurement
	5	Linear slope of the last six weight-for-height percentile measurements
	6	Weight percentile at 4th measurement
	7	Weight-for-height percentile at the fifth measurement
	8	Weight percentile at the fifth measurement
	9	Linear slope of the last six weight percentile measurements

Footnote: Measurement order refers to the chronological sequence of recorded growth measurements within the relevant age window. For example, “4th measurement” indicates the fourth recorded visit during that period. “Higher of the last two measurements” refers to the higher value of the two most recent recorded percentiles. “Maximum percentile” represents the highest observed weight-for-length (or weight-for-height) percentile during the specified age window.

**Table 3:** The models’ performance at different cut points.

Model	Cut Point	Sensitivity	Specificity	PPV	PPV85 *	NPV	Cumulative Lift
0–3 months	99	7%	99%	43%	77%	95%	7.3
	95	27%	95%	26%	64%	96%	4.4
	90	40%	91%	21%	57%	96%	3.6
	85	48%	86%	17%	52%	96%	3.0
3–6 months	99	9%	100%	54%	86%	95%	9.3
	95	33%	96%	31%	73%	96%	5.4
	90	48%	91%	25%	66%	97%	4.3
	85	58%	87%	21%	60%	97%	3.6
6–12 months	99	11%	100%	64%	94%	95%	10.8
	95	41%	96%	40%	83%	96%	6.8
	90	57%	92%	31%	75%	97%	5.2
	85	67%	87%	25%	69%	98%	4.2

Footnote: Model performance metrics are presented for the prediction of early childhood overweight, defined as weight-for-length (or weight-for-height) ≥97th percentile according to WHO standards at age 18–36 months. Thresholds correspond to percentile-based risk cutoffs derived from the model risk scores. * Calculation of positive predictive value under the assumption that the prediction is correct in case the individual has weight/length percentile equal or higher than 85 (instead of 97).

## Data Availability

The deidentified database contains the medical records of Israeli children that support the findings of this study, which were used under license from the Israeli Ministry of Health for the current study, and may be available for other under a similar license.

## Abbreviations

WHO—World Health Organization; AUC—Area Under the ROC Curve; CI—Confidence Interval; IOM—Institute of Medicine; AAP—American Academy of Pediatrics; MOH—Ministry of Health; HMO—Health Maintenance Organization; THIS program—Tipat Halav Israeli Screening program; EPDS—Edinburgh Postnatal Depression Scale; PPV—Positive Predictive Value; NPV—Negative Predictive Value; BMI—Body Mass Index; EMR—Electronic Medical Record.

## Appendix A

**Table A1:** AUC of the three models for children using the last measurements at 18–24 months and 24–36 months separately.

Model	Target: 18–24 Months		Target: 24–36 Months	
	ML Model	WHO	ML Model	WHO
0–3 months	77.12%	69.43%	75.81%	68.61%
3–6 months	83.91%	81%	80.96%	77.50%
6–12 months	89.27%	88.43%	85.55%	84.64%

Footnote: Values are presented separately for each model according to age-specific inclusion criteria (0–3, 3–6, and 6–12 months). Differences in sample size reflect the availability of growth measurements within each age window and model-specific eligibility requirements.
